# Acute threat enhances perceptual sensitivity without affecting the decision criterion

**DOI:** 10.1038/s41598-022-11664-0

**Published:** 2022-05-31

**Authors:** Lycia D. de Voogd, Eline Hagenberg, Ying Joey Zhou, Floris P. de Lange, Karin Roelofs

**Affiliations:** 1 Donders Institute for Brain Cognition and Behaviour, Centre for Cognitive Neuroimaging, Radboud University, Kapittelweg 29, 6525 EN Nijmegen, The Netherlands; 2 Behavioural Science Institute, Radboud University, Nijmegen, The Netherlands

**Keywords:** Human behaviour, Cognitive neuroscience, Emotion, Visual system

## Abstract

Threatening situations ask for rapid and accurate perceptual decisions to optimize coping. Theoretical models have stated that psychophysiological states, such as bradycardia during threat-anticipatory freezing, may facilitate perception. However, it’s unclear if this occurs via enhanced bottom-up sensory processing or by relying more on prior expectations. To test this, 52 (26 female) participants completed a visual target-detection paradigm under threat-of-shock (15% reinforcement rate) with a manipulation of prior expectations. Participants judged the presence of a backward-masked grating (target presence rate 50%) after systematically manipulating their decision criterion with a rare (20%) or frequent (80%) target presence rate procedure. Threat-of-shock induced stronger heart rate deceleration compared to safe, indicative of threat-anticipatory freezing. Importantly, threat-of-shock enhanced perceptual sensitivity but we did not find evidence of an altered influence of the effect of prior expectations on current decisions. Correct target detection (hits) was furthermore accompanied by an increase in the magnitude of this heart rate deceleration compared to a missed target. While this was independent of threat-of-shock manipulation, only under threat-of-shock this increase was accompanied by more hits and increased sensitivity. Together, these findings suggest that under acute threat participants may rely more on bottom-up sensory processing versus prior expectations in perceptual decision-making. Critically, bradycardia may underlie such enhanced perceptual sensitivity.

## Introduction

Acutely threatening situations often require rapid decisions based on limited available perceptual information. Such acutely uncertain threatening situations trigger fast autonomic changes that prepare the body to freeze, fight or flight. Theoretical models have stated that affective states such as freezing may facilitate decision-making under uncertainty for example by enhancing threat processing and detection^1,2^ and value-based decision-making^3^. Threat-induced affective states may therefore increase bottom-up processing of sensory stimuli and improve perceptual sensitivity under threat^4–8^. Moreover, facilitation of sensory processing also occurs via top-down expectations e.g.^9–13^. Indeed, it may be adaptive to take previously learned information into account when making decisions under threat. Whether threat simultaneously or selectively influences bottom-up and top-down processes is unclear.

Uncertain perceptual decisions involve an inferential process where both bottom-up sensory input and top-down prior knowledge or expectations are weighted^9^. Namely, when sensory information is weak participants tend to show a bias in their decisions^14^. However, this could lead to perceptual errors and in threatening situations these errors can have drastic consequences^15^. An optimal weighing of bottom-up sensory input and top-down prior expectations is crucial to make a correct decision in threatening situations.

Previous studies have indicated that threat enhances perceptual sensitivity^4–8^. For example, human participants were more accurate at judging the orientation of a grating when it was cued by a fearful face compared to a neutral one^6^. Moreover, detection of low contrast gratings was enhanced when those gratings were previously paired with an aversive stimulus compared to when they were not^7^. Two other studies^4,5^ additionally found that while being under the threat of receiving an electrical shock, grating orientation judgements are specifically improved when the gratings consist of a low spatial frequency (i.e. 3 cycles per degree) compared to a higher spatial frequency (i.e. 6 cycles per degree). Critically, this was linked to bradycardia, typical for threat-anticipatory freezing. Freezing is characterized by concurrent sympathetic and parasympathetic upregulation, with dominance in the cholinergically-driven parasympathetic branch of the autonomic nervous system (ANS) resulting in a net heart rate deceleration. This is the case even when sympathetic indices such as skin conductance show upregulation. Only bradycardia and not skin conductance was related to the enhanced sensitivity to coarse visual feature^5^, suggesting that specifically threat-anticipatory freezing states may underlie upregulation of bottom-up sensory input.

Animal models indicated that the amygdala, a region critically implicated in threat detection and cardiac and behavioural threat responses^16^, has projections to sensory cortical areas^17–19^. The amygdala may facilitate perception via those feedback connections^20^. In line with this, human studies have found increased BOLD responses in the visual cortex^4,7^ as well as increased connectivity between the amygdala and the visual cortex during threat compared to safe^4^. Together, these findings suggest that threat-induced psychophysiological states enhance bottom-up sensory processing during perceptual decision-making.

However, these studies did not investigate whether acute threat also influences the use of top-down prior expectations when making perceptual decisions. Moreover, enhanced sensitivity can also be a result of enhanced bottom-up processing. Threat-related processes can increase the reliance on previously learned information. For example, after a stress induction, participants exhibited increased habitual stimulus–response behaviour compared to goal-directed action-outcome behaviour^21^. Moreover, theoretical models have stated a common underlying “better-safe-than-sorry” processing strategy in threat-related psychopathology^22^. As an example, one study found that participants scoring high on neuroticism show greater avoidance behaviour for ambiguous stimuli that could potentially predict a shock^23^. Another study found that individuals who score high on trait anxiety showed increased usage of prior information on current decisions^24^. However, whether acute threat influences the reliance on prior expectations during perceptual decision-making remains to be investigated.

In the present study we aimed to investigate the influence of acute threat on perceptual decision-making by disentangling the influence of threat on bottom-up sensory processing and on use of prior expectations. Participants completed a visual target-detection paradigm (﻿adapted from:^25^) under threat-of-shock or safety, with a manipulation of prior expectations. Half of the trials were indicated as threat-of-shock by the colour of the fixation dot (15% reinforcement rate). Participants judged the presence of a backward-masked grating (target presence rate 50%) after manipulating their decision criterion with a rare (20%) or frequent (80%) target presence rate procedure. First, we predicted that the number of correctly identified targets (i.e. hits) and perceptual sensitivity (i.e. *d*’) would increase under threat-of-shock compared to the safe condition^4–8^. Second, we tested the hypothesis whether threat would affect top-down processing by manipulating the decision criterion. Such a threat influence on the reliance of prior expectations could occur in two possible ways. The first option is that regardless of the target frequency manipulation (Rare, Frequent) the overall criterion would be lower, meaning participants would have an increased tendency to indicate the target is present for the threat-of-shock compared to the safe condition (i.e. a liberal response bias). This would be in line with the better-safe-than-sorry heuristic^22^. The second option is that under threat-of-shock (compared to safe), participants would rely more on the prior expectation and their decision criterion would follow the target frequency manipulation. Third, we expected a stronger heart rate deceleration under threat-of-shock compared to safe, which would be related to the change in perceptual decision-making. Namely, within-subjects we expected stronger heart rate deceleration for correctly identified targets versus missed targets that may be enhanced under threat.

## Results

### Target-frequency manipulation alters decision criterion

**Figure 1:**
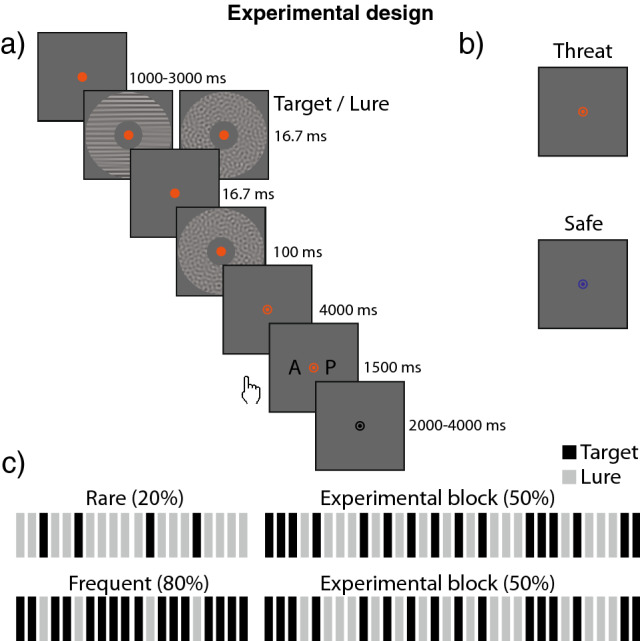
(**a**) Trial structure during the experimental blocks (**b**) orange and blue fixation colour indicating the threat-of-shock or safe condition (counterbalanced across subjects) (**c**) structure of the target-frequency manipulation, in black is the occurrence of the target and grey the lure.

We first verified whether our target-frequency manipulation (Rare, Frequent) induced the expected criterion shift (i.e. the tendency to report a target was present). As expected, the criterion was higher following a target detection block in which the target was only present 20% of the time (Rare; M = 0.53, SD = 0.37) compared to when the target was present 80% of the time (Frequent; M = 0.20 SD = 0.49) [Z = −4.03, *p* < 0.001, r = 0.56, 95% CI (0.37, 0.76)]. This means that participants were less likely to state the target was present following the Rare target blocks compared to the Frequent target block. Our paradigm was therefore successful in inducing a criterion shift. See Fig. 1 for the experimental design.

### Threat-induced psychophysiological changes

**Figure 2:**
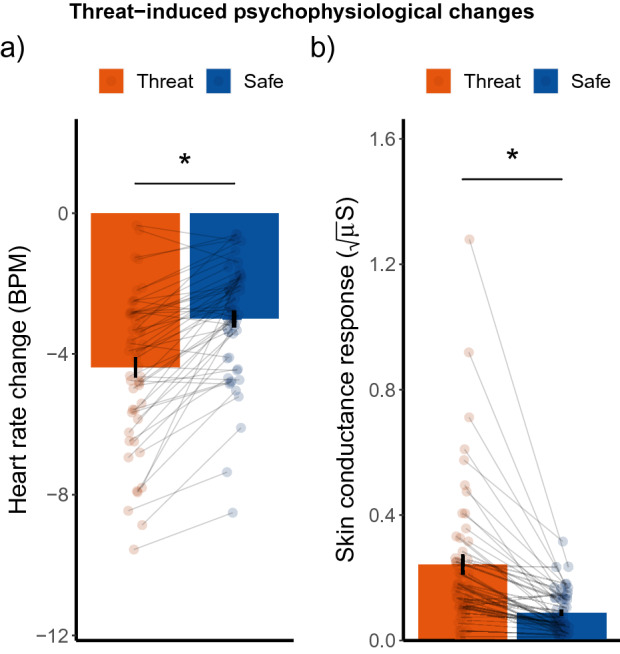
(**a**) The average heart rate change (i.e. baseline corrected) in beats per minute (BPM) and (**b**) Skin conductance responses (SCR)—during threat-of-shock and safe trials. Error bars represent + /− standard error of the mean (SEM).

We next verified whether our threat-of-shock manipulation was effective in inducing physiological changes typical for threat-anticipatory freezing. This includes parasympathetic dominance (net heart rate (HR) decrease; Threat: M = −4.38 SD = 2.12, Safe: M = −2.99 SD = 1.68) with concurrent sympathetic upregulation (skin conductance response (SCR) increase; Threat: M = 0.24 SD = 0.24, Safe: M = 0.09 SD = 0.07). Accordingly, the magnitude of the average HR deceleration [t(51) = −7.39, *p* < 0.001, d = −1.03, one-tailed, 90% CI (−1.23, −0.77)] and magnitude of the SCR [t(51) = 5.52, *p* < 0.001, d = 0.77, one-tailed, 90% CI (0.53, 0.91)] were was significantly higher during threat-of-shock compared to safe trials. Our paradigm was therefore successful in inducing threat-related physiological changes. See Fig. 2.

### Threat increases sensitivity but no evidence for changes in the response bias

**Figure 3:**
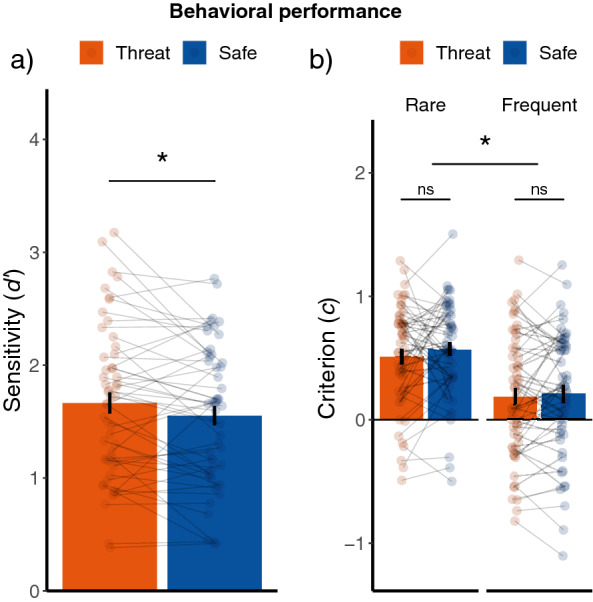
(**a**) Increased sensitivity (d’) under threat-of-shock (**b**) no interaction between threat-of-shock manipulation and the target-frequency manipulation on the decision criterion. Error bars represent + /− standard error of the mean (SEM).

We then tested our primary hypothesis that threat would enhance perceptual sensitivity. We indeed found that the number of hits [Threat: M = 0.68 SD = 0.14, Safe: M = 0.65 SD = 0.13; t(51) = 2.57, *p* = 0.007, d = 0.36, one-tailed, 90% CI (0.08, 0.60)] as well as the *d’* [Threat: M = 1.66 SD = 0.68, Safe: M = 1.55 SD = 0.62; Z = −2.30, *p* = 0.021, r = 0.32, one-tailed, 90% CI (0.13, 0.50)] was higher during threat-of-shock trials compared to safe trials. Thus, in line with our expectations, acute threat increased perceptual sensitivity. See Fig. 3a.

Lastly, we tested whether acute threat would affect the response bias which could occur in either of two possible ways. We did not find evidence for this: the criterion did not significantly differ between threat-of-shock and safe trials [Z = -1.07, *p* = 0.28, r = 0.10, 95% CI (-0.08, 0.35)]. Moreover, the threat-of-shock manipulation also did not significantly interact with the target-frequency manipulation [Rare Threat: M = 0.51 SD = 0.42, Rare Safe: M = 0.57 SD = 0.40, Frequent Threat: M = 0.19 SD = 0.51, Frequent Safe: M = 0.21 SD = 0.51; Z = −0.82, *p* = 0.41, r = 0.08, 95% CI (−0.12, 0.29)], so we did not find evidence that threat-of-shock changed the influence of the target-frequency manipulation on the response bias. See Fig. 3b.

Together, these findings indicate that threat enhances perceptual sensitivity, but we did not find evidence for the hypothesis that threat-of-shock alters reliance on prior expectations.

### Association between sensitivity and response bias

We additionally verified whether there was a relationship between sensitivity and response bias as a function of our target-frequency manipulation. It is possible that when sensory information becomes stronger (as indexed by a higher *d’*), biases in decision making become weaker. Indeed, there was a negative correlation, across participants, between *d’* and the difference in response bias between the manipulation types [ρ(50) = −0.29, *p* = 0.04, 95% CI (−0.56, −0.02)]. This means that higher sensitivity was accompanied with less influence of the target-frequency manipulation on the decision criterion.

### Heart rate deceleration associated with correct target detection

**Figure 4:**
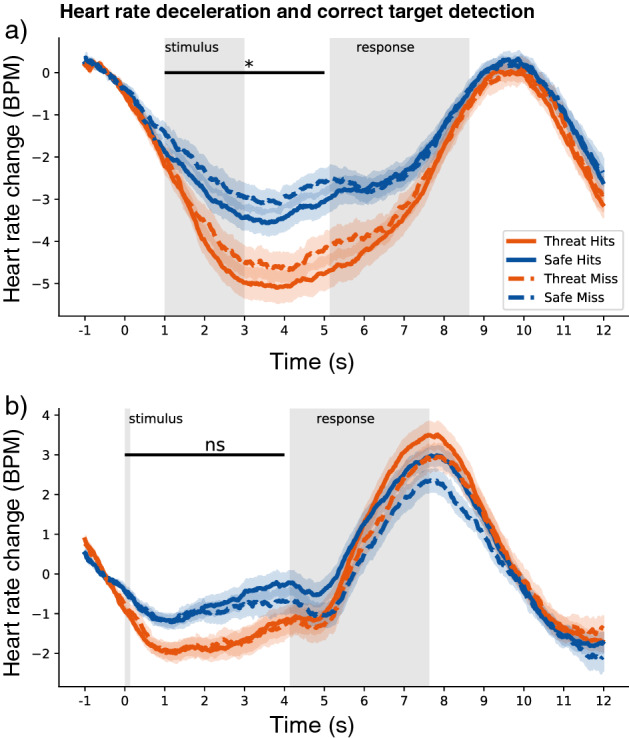
When aligned to the onset of the trial (which indicated the threat-of-shock or safe trial) there was an (**a**) increased heart rate deceleration for correctly identified targets versus missed targets for both threat-of-shock and safe trials. This significant difference was not present (**b**) when heart rate was aligned to the target onset. Shaded area represents standard error of the mean (SEM).

Next, we tested whether there was an association between the threat-induced physiological changes and increased sensitivity under threat. We found a stronger heart rate deceleration when participants correctly detected targets (i.e. hits) compared to when they did not report seeing a target (i.e. misses) [F(1, 51) = 5.33, p = 0.025, petasq = 0.09, 95% CI (−0.01, 0.19)]. Such a relationship between target detection and SCRs was not significantly present [F(1, 51) = 1.00, *p* = 0.32, petasq = 0.02, 95% CI (−0.05, 0.07)]. The increase in heart rate deceleration for hits was, however, not more pronounced during threat-of-shock compared to safe trials [F(1, 51) = 0.04, *p* = 0.85, petasq < 0.01, 95% CI (−0.05, 0.03)]. Even though there were more hits (and less miss trials) and a higher *d’* for threat-of-shock compared to safe trials, the association between heart rate deceleration and target detection was present for both conditions. See Fig. 4a.

To rule out the possibility that the heart rate deceleration was *induced* by the target detection rather than a *consequence* of the heart rate deceleration, we investigated whether heart rate deceleration was different between hits and misses following the stimulus when taking the onset of the stimulus into account rather than the start of the trial. Please note that the stimulus onset varied between 1000 and 3000 ms from the start of the trial. We found no significant difference in heart rate deceleration between hits and misses when taking the onset of the stimulus into account [F(1, 51) = 0.80, *p* = 0.38, petasq = 0.02, 95% CI (−0.06, 0.06)]. See Fig. 4b.

Finally, we verified whether heart rate deceleration (relative to baseline) during safe trials could reflect a trial-to-trial carryover effect from previous threat-of-shock trials. If heart rate deceleration during safe trials reflects a carryover effect from previous threat-of-shock trials, one would expect lower heart rate deceleration during safe trials that followed a threat-of-shock trial compared to safe trials that followed a safe trial. However, there was no significant difference in heart rate deceleration between these two types of safe trials [t(51) = -1.67, p = 0.10, d = -0.23, 95% CI (-0.50, 0.04)].

In conclusion, heart rate deceleration was associated with correct target detection. This association was not more pronounced under acute threat despite the increase in correct target detections.

### Heart rate deceleration associated with faster reaction times

**Figure 5:**
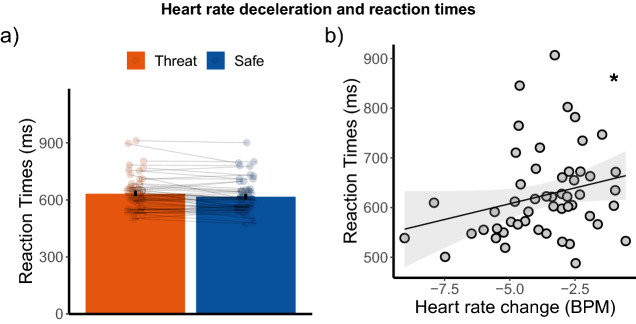
(**a**) Slower reaction times during threat-of-shock compared to safe trials (**b**) heart rate deceleration associated with faster reaction times which did not significantly differ between threat-of-shock and safe trials. Error bars represent + /− standard error of the mean (SEM).

To better understand the association between heart rate deceleration and correct target detection, we checked whether heart rate was also associated with reaction times. Please note there was a delay of 4 s between the stimulus and the response window, therefore the paradigm did not involve speeded decisions. Nevertheless, we found that reaction times (on both hit and miss trials) were slower on threat-of-shock [M = 632.4, SD = 92.1] trials compared to safe [M = 616.5, SD = 88.4] trials [Z = −2.76, *p* = 0.006, r = 0.38, 95% CI (0.15, 0.62)]. Across participants, the difference in reaction times between threat-of-shock and safe trials were not significantly correlated with the difference in heart rate deceleration between threat-of-shock and safe trials [ρ(50) = −0.16, p = 0.25, 95% CI(-0.43, 0.09)]. However, the average heart rate deceleration across participants was correlated with the average reaction time [ρ(50) = 0.32, p = 0.02, 95% CI(0.05, 0.59)]. Namely, participants with on average stronger heart rate deceleration responded on average faster. See Fig. 5.

## Discussion

In this study we investigated whether acute threat influences perceptual decision-making via enhanced bottom-up sensory processing or by relying more on prior expectations. Participants completed a visual target-detection paradigm under threat-of-shock (15% reinforcement rate) with a manipulation of prior expectations. We found that threat-of-shock induced stronger heart rate deceleration and increased SCRs compared to safe, indicating that our paradigm was successful in inducing a threat-related physiological state. Importantly, threat-of-shock enhanced perceptual sensitivity (*d’*), but we did not find evidence for alterations in the decision criteria. Correct target detection (hits) was furthermore accompanied by an increase in the magnitude of this heart rate deceleration compared to a missed target. While this was independent of threat-of-shock manipulation, only under threat HR bradycardia -indicative of parasympathetic dominance- was accompanied by more hits and increased sensitivity.

We found enhanced perceptual sensitivity (*d’*) under threat in a visual target-detection paradigm. This finding is in line with previous studies showing enhanced perceptual sensitivity (*d’*) under threat in visual grating orientation discrimination paradigms^4–8^. We extend previous findings by showing acute threat can enhance target detection, a process that occurs earlier than orientation judgements and much more rapidly by a pre-attentive process that serves to detect changes in relation to the environment^26^. Moreover, we have used a backward masking procedure which interferes recurrent processes required for object recognition. Despite this, we still found enhanced sensitivity under threat-of-shock. Orientation discrimination relies on serial search of the positional relationship between the parts that make up the stimulus by focal attention^27^. Evidence for a distinction between these two processes comes from the finding that in monkey primary visual cortex, neuronal activity reflecting pre-attentive motion detection appeared significantly earlier compared to activity reflecting selective attention^28^. Moreover, orientation judgements rely on a working memory process^29^, because the first orientation has to be kept in working memory to be able to perform the comparison. Therefore, the threat-enhanced perceptual sensitivity found in previous studies could be due to an influence of threat on working memory processes. Here we show that acute threat already influences perceptual sensitivity at early stages of visual processing.

Although we expected that heart rate deceleration would specifically be related to enhanced target detection under acute threat, we found this was also the case in the safe condition. Hits, compared to misses, in both the threat-of-shock and safe condition were associated with increased heart rate deceleration. How can we explain this finding? Heart rate deceleration has robustly been found during threat anticipation (e.g.^4,5,30,31^). However, such deceleration is not specific for threat-related processes. Heart deceleration has been associated with attention and action preparation. For example, heart rate deceleration has been found during the anticipation of a target stimuli presented at an attended location compared to an unattended location^32^. Interestingly, attention was shown to increase the weight given to sensory evidence^10^. Therefore, it is possible that fluctuations in attention across the experiment are linked to improved detection of the stimulus^33,34^. With greater attention, indicated with stronger heart rate deceleration, resulting in more hits as opposed to a miss regardless of the threat condition. Similarly, in cases when an action is required compared to no action heart rate deceleration is observed during an anticipation window^31^. As action preparation is associated with enhanced perception of task-relevant features of objects^35^, it is also possible that increases in action preparation can explain the increase in hits. Indeed, we found across participants, decreased heart rate was associated with faster reaction times. This is in line with previous studies showing such an association^30,36^. This notion is in line with the hypothesis that freezing acts as a brake on the already activated motor system facilitating a fast switch to action by simply releasing the parasympathetic break^1,3^.

Importantly, even though we found increased heart deceleration for hits compared to misses for both threatening and safe conditions, only under acute threat this led to more hits (and better perceptual sensitivity when taking false alarms into account). Although we are unable to conclude this from our study, it is possible that under acute threat there is an upregulation of sensory areas through amygdala projections^4,17–19^. Lojowska et al. (2018) found a relation between threat-related heart rate deceleration and threat-related BOLD responses in V1. A possible mechanistic explanation could be that this upregulation is driven by cholinergic projections from the amygdala to visual cortical areas. It has been shown that cholinergic activation of the visual cortex is linked to enhanced visual perception (for a review see^37^). Moreover, administration of a cholinergic agonist was found to enhance visual attention via modulation of the visual cortex in humans^38^. Importantly, cholinergic activation of the basolateral amygdala has also been linked to freezing behaviour in rodents^39^. Threat-induced freezing states may therefore potentially lead to a cholinergic-driven increase in amygdala-visual cortex coupling. This pairing of cholinergic activation with visual stimulation may then increase signal-to-noise ratio in the visual cortex and improve perceptual sensitivity^37^.

If this is the case then threat-induced freezing should increase neural specificity for visual stimuli encoded under threat. In line with this, two studies^4,7^ found indeed enhanced visual cortex activation under threat that was linked to behavioural performance. However, they did not find this increase in activation was stimulus specific. A possible explanation for this could be that the visual stimuli that needed to be judged were not relevant to the aversive outcome. Indeed, it is the affective significance of a visual information that is thought to lead to enhanced visual processing^6,33^. As increased signal-to-noise distributions can be extracted from activation patterns in the visual cortex e.g. see^40^, future neuroimaging studies could use analyses to investigate this in a design in which the visual information is relevant to the aversive outcome. Moreover, a future study using cholinergic antagonist administration (e.g. scopolamine) could reveal cholinergic involvement in the gain in neural specificity by blocking threat-induced perceptual sensitivity.

Our paradigm was successful in inducing a criterion-shift (replicating Zhou et al.^25^). However, we did not find evidence for a threat-induced change in criterion shift in any specific direction. More specifically, we did not find evidence that acute threat increases or decreases the reliance on prior expectations, nor did we find evidence for a better-safe-than-sorry approach. First, it is possible that the enhanced perceptual sensitivity during threat may have reduced the influence of threat on the decision criterion. As the perceptual sensitivity and decision criterion may not be completely orthogonal this could be a plausible explanation. If sensory information becomes stronger, biases in decision making become weaker^13^. Indeed, we observed that, across participants, sensitivity was negatively correlated with the difference in response bias between the two target-frequency manipulation conditions. Second, our paradigm (using the 3-down-1-up staircase procedure) may not have led to enough sensory uncertainty. However, the observed criterion-shift due to the target-frequency manipulation indicated there was sufficient sensory uncertainty and renders this alternative hypothesis unlikely. Third, our design did not evolve speeded decisions. Another possibility for the absence of a significant difference in criterion-shift under acute threat compared to safe, is therefore that the reliance on prior expectations may only occur when there is limited time to decide. We cannot rule out that the delay between the stimulus and the response (i.e. 4 s) may have strengthened the processing of sensory information or weakened the reliance on prior expectations. Fourth, our design did not involve the targets to be relevant to the aversive outcome. Because the affective significance of visual information is thought to lead to enhanced visual processing^6,33^, we cannot rule out the absence of a direct association between the target and the aversive outcome influenced the reliance on prior expectations as well. Indeed, attention and bias modulation could be dependent on the saliency of the stimuli (e.g.^8,41^). Nevertheless, future studies could systematically manipulate sensory uncertainty, decision time, and threat-outcome relevance in addition to manipulations of prior expectations and threat.

In conclusion, we found that acute threat enhanced perceptual sensitivity in a visual target-detection paradigm. However, there was no evidence for a threat-induced alteration of the prior expectation on current decisions. Our findings are in line with theoretical models^1–3^ on how freezing state may facilitate accurate approach-avoid decisions by improved perceptual decisions. Together, these findings suggest that under acute threat participants may rely more on bottom-up sensory processing versus prior expectations in perceptual decision-making. Critically, psychophysiological states, such as freezing-related bradycardia, may underlie such enhanced perceptual sensitivity.

## Methods

### Participants

In total, 55 participants completed the entire study. One additional participant terminated the experiment early. From the 55, 3 participants were excluded from the analyses: one participant did not comply with the instructions (i.e. > 33% non-responses), two participants were excluded due to floor and ceiling performance on the task as for those participants the staircase procedure was not effective. This was indicated by a deviation in performance of more than 2 SD from the mean (< 57% and > 93%). This resulted in a final sample of 52 (26 females, Age: *M* = 25, SD = 7.8, 8 left-handed) participants. Sample size was determined based on a medium effect size (mean difference between two matched pairs), Cohen’s dz = 0.40, alpha = 0.05, and 1—beta = 0.80 using G*Power^42^. Data collection was finished when 52 participants could be included in the analysis. The effect size was determined by the difference in heart rate between threat-of-shock and safe in Lojowska et al.^4^ and difference in criterion between the rare and frequent target presence rate in Zhou et al.^25^. Exclusion criteria for participation were as follows: age (< 16 years old), claustrophobia, current pregnancy, not speaking English, uncorrected vision, colour-blindness, habitual smoking, average use of > 3 alcoholic beverages daily, average use of recreational drugs, current treatment with any medication. All participants gave written informed consent before participation, and were paid for participation (€20). All research activities were carried out in accordance with the Declaration of Helsinki and approved by the local ethics committee: Commissie Mensgebonden Onderzoek (CMO) / Medisch Ethische Toetsings Commissie (METC) Arnhem-Nijmegen, *CMO 2014/288*) and conducted according to these guidelines and regulations.

### Experimental design and procedure

Participants completed a visual target-detection paradigm with a manipulation of prior expectations^25^ under a threat-of-shock and safe condition. The task of the participant was to indicate as accurately as possible whether a target grating was present or absent. In case the target was absent, a lure was presented. Participants first completed a standardized 5-step shock work-up^43^ to calibrate the intensity of the shocks used throughout the experiment. Next, participants were instructed on the paradigm and performed a familiarisation block followed by a 3-down-1-up staircase procedure^44^. The goal of the staircase procedure was to titrate performance  ~ 75% mean accuracy (as was found in^25^ using a similar design) by increasing the contrast of the backward mask such that visibility of the target was reduced. The contrast of the target remained the same. Subsequently, participants performed the visual target-detection paradigm which was divided into 8 blocks with short breaks in between. Each block consisted of a criterion-shift manipulation block (20 trials ~ 2 min duration) during which the target presence rate either 20% (Rare condition) or 80% (Frequent condition) and an experiment block (38 trials, ~ 6 min 30 s duration) during which the target was presented 50% of the time. During the experimental block, the colour (Orange or Blue: counterbalanced across participants) of the fixation dot indicated whether the participant was in the threat-of-shock or the safe condition. Half of the trials were indicated as a threat-of-shock trial, of which only 15% (3 out of 20) were reinforced (note that those reinforced trials were not included in the analysis). The shock could occur at any moment during a trial (except the ITI). Participants were instructed to remain fixated at the fixation bulls-eye and always give a response, even if they were not sure whether they saw the target or not. The experiment was programmed using the Psychophysics Toolbox (Brainard, 1997) in Matlab (Matlab, R2020a, The Mathworks, Inc.) and ran in a Linux environment. The stimuli were presented on a gamma-corrected LCD screen (1920 × 1080 pixel resolution, 60-Hz refresh rate).

### Familiarisation and staircase procedure

Participants first got acquainted with the paradigm through a familiarisation block. This block was designed so the target was clearly visible by having a low contrast backward mask and longer duration (i.e. first 10 trials) between the target and the lure. There were 10 trials where a fixation dot was presented (500 ms), followed by the target or lure (16.7 ms), an ISI period (200 ms), the backward mask (100 ms), an ISI period (800 ms), a response period (1500 ms) and the ITI (2000 ms). These trials were followed by 20 trials where this ISI was reduced from 800 ms to 16.7 ms, which remained throughout the rest of the experiment. Prior to this familiarisation block, participants were informed through on-screen instructions that the target presence rate was 50% and that they would receive feedback on their performance. Feedback was indicated with a green ‘plus’ sign for correct responses or a red ‘minus’ sign for incorrect responses in place of the fixation bull’s eye at the end of each trial.

Immediately following the familiarisation block, a staircase block was completed for each of two target orientations (90° and 180° from horizontal) separately. To ensure participants were unaware of the purpose of the staircase procedure, participants were solely informed that they would continue practicing without feedback and it would be more difficult (i.e. the durations were the same as in the experimental blocks, see below). During the staircase procedure the Michelson contrast of the mask was titrated to get an overall mean accuracy of ~ 75%, (as in^25^). The resulting average accuracy in this experiment was M = 75% (SD = 8%). Participants received explicit instructions on both the presence rate (50%) and the orientation of the target prior to each staircase block. The staircase blocks consisted of 40 trials each with the same durations as the familiarisation block. The starting contrast for the backward mask was 60%, which would increase after three consecutive correct responses and decrease after an incorrect response.

At the end of the staircase blocks, an average contrast was calculated based on all except for the first three reversals. In addition, the contrast changes during the staircase were plotted and visually inspected. The prerequisite for a successful staircase procedure was that the contrast at the end of the procedure would converge around the calculated average. If at least one of the graphs did not converge, but instead either showed a downwards or upwards trend, both staircase blocks would be started over with a starting contrast of 50% or 70%, respectively. The resulting average contrast was set throughout the rest of the paradigm (*M* = 51%, SD = 15%).

### Visual target-detection paradigm under threat-of-shock

The paradigm consisted of eight blocks, each consisting of a criterion-shift manipulation block and an experimental block consecutively.

During the target-frequency manipulation (Rare, Frequent) the backward mask had a contrast of 5% Michelson contrast to increase target visibility and therefore the efficacy of the manipulation. Four blocks had a target presence rate of 20% (Rare condition) and four had a target presence rate of 80% (Frequent condition). Participants were instructed about this presence rate. The order was counterbalanced across participants. The target-frequency manipulation consisted of 20 trials each, where each trial consisted of a fixation period (500 ms), followed by the target or lure (16.7 ms), an ISI period (16.7 ms), the backward mask (100 ms), an ISI period (800 ms), a response period (1500 ms) and the ITI (2000 ms). Importantly, the fixation dot was black and participants were instructed they could not receive a shock. The reason for this was to make sure the learning of the target-frequency manipulation itself was not influenced by threat-of-shock.

The target-frequency manipulation (Rare, Frequent) was followed by the experimental block, which consisted of 32 trials (16 threat, 16 safe). There were six additional trials that were not included in the analysis. Three were target trials and three lure trials to ensure the 50% target rate. Four of the six were threat-of-shock trials, of which three were reinforced (i.e. 15% reinforcement rate), and two were safe trials. In addition, to ensure attentiveness, four of the six trials included a response period that was shortened (800—3000 ms). The reason these six trials were not included in the analysis was because they either included a shock or the response period was not long enough to measure the heart rate deceleration. The order of the 38 trials was randomised. Further, each trial commenced with the fixation colour that was either orange or blue (1000–3000 ms). This was followed by the presentation of the target or lure (16.7 ms), an ISI period (16.7 ms), the backward mask (100 ms), an ISI period (4000 ms), a response period (1500 ms) and the ITI (2000—4000 ms). During the response period and the ITI, the fixation colour was changed back to black. In contrast to the target-frequency manipulation, the target presence rate during the test blocks was always 50%. Participants were not instructed about this change.

### Stimuli

Each trial contained a bull’s eye fixation dot consisting of an inner dot (0.25° × 0.25° degree visual angle; dva) and an outer ring (0.5° × 0.5° dva). Depending on the trial type this fixation bull’s eye was black (RGB: 255, 255, 255), orange (RGB: 255, 127.5, 0) or blue (RGB: 0, 127.5, 255). The first stimulus was either a target (grating with a horizontal or vertical orientation, Michelson contrast: 40%, spatial frequency: 1 cycle per degree (cpd), randomised spatial phase) or a lure (bandpass-filtered noise-patch, Michelson contrast: 40%, spatial frequency: 1 cpd, randomly generated for each trial). The second stimulus was another noise-patch that served as a backward mask (bandpass-filtered noise patch, spatial frequency: 1 cpd, randomly generated for each trial, inner radius = 1.5°, outer radius = 10°, contrast decreased linearly over the inner and outer 0.5° of the annulus). The Michelson contrast of this noise-patch was set during the staircase procedure. The stimuli were presented in an annulus centred around the fixation dot (inner radius = 2°, outer radius = 10°, contrast of the stimuli decreased linearly to 0 over the inner and outer 0.5° of the annulus). During the response period, the letters ‘P’ (for target present) and ‘A’ (for target absent) were presented on each side of the bull’s eye (5° dva), indicating the response mapping of the current trial. The location of each letter (left or right) changed pseudo randomly across trials to avoid motor preparation confounds.

### Behavioural data and analysis

As our primary behavioural outcome variables we calculated the *d’* [*d'* = z(Hit rate)-z(False Alarm rate)] and criterion [−1/2 (z(Hit rate) + z(False Alarm rate))] based on hit rates and false alarm rates. A correction factor of 0.25 was used in the case of no false alarms or a perfect hit rate to allow for a *d’* or criterion calculation. Trials where participants did not respond or were too late (> 1500 ms) were not included in the analysis [M = 1.35, SD = 2.19%]. To test our primary hypothesis, we calculated a *d’* for the threat-of-shock and safe condition separately. For our two hypotheses regarding the criterion, we calculated a criterion first for Threat (Threat, Safe) and then for four conditions Threat (Threat, Safe) X Target frequency (Rare, Frequent) separately. As an additional analysis, we also tested for reaction time differences between the threat and safe condition. We used a non-parametric Wilcoxon signed-rank test on *d’* and criterion to minimize any potential non-normality and outlier concerns due to the nature of the calculation of these measures, and on RTs. For the criterion analysis, we additionally verified the outcome of the separate Wilcoxon signed-rank tests using a aligned-rank-transform (ART) procedure (i.e. a non-parametric approach to factorial ANOVA^45^). This yielded similar outcomes, therefore the Wilcoxon signed-rank tests will be reported.

### Peripheral measurements and analysis

Finger pulse and electrodermal activity was assessed using a BrainAmp MR system and recorded using BrainVision Recorder software (Brain Products GmbH) with a sample frequency of 5000 Hz.

Finger pulse was recorded using a pulse oximeter affixed to the fourth distal phalanx of the non-dominant hand. After downsampling to 50 Hz, finger pulse data was pre-processed using PulseCor (https://github.com/lindvoo/PulseCor) implemented in Python 3.7. Pre-processing automatic peak detection with addition manually inspection and correction. For analyses, the average beats per minute (BPM) was calculated between a time window of 1 to 5 s following trial onset, corrected for a baseline period of 1 s prior to trial onset. This time period was chosen so it did not include button presses. To test whether our threat-of-shock manipulation was successful we performed a paired t-test with Threat (Threat, Safe) as a within-subject variable on BPMs. In addition, to investigate an association between the threat-of-shock manipulation and target detection we performed a repeated-measures ANOVA with Threat (Threat, Safe) and Target detection (Hit, Miss) as a within-subject variable.

Electrodermal activity was assessed using two Ag/AgCl electrodes attached to the distal phalanges of the second and third finger of the non-dominant hand. After downsampling to 250 Hz, skin conductance responses (SCRs) were automatically scored with additional manual supervision using Autonomate^46^ implemented in MATLAB R2020a (The MathWorks, Inc). SCR amplitudes (measured in microsiemens) were determined for each trial within an onset latency window between 0.5 and 5.133 s after stimulus onset, with a minimum rise time of 0.5 s and a maximum rise time of 5 s after response onset. In case of multiple SCR responses meeting these requirements, the largest response was used for analysis. All amplitude differences were square root transformed prior to statistical analysis. To test whether our threat-of-shock manipulation was successful we performed a paired t-test with Threat (Threat, Safe) as a within-subject variable on SCRs.

### Peripheral stimulation

Electrical shocks were delivered via two Ag/ AgCl electrodes attached to the distal phalanges of the fourth and fifth finger of the dominant hand using a MAXTENS 2000 (Bio-Protech) device. Shock duration was 200 ms, and intensity varied in 10 intensity steps between 0–40 V/0–80 mA. During a standardized shock intensity adjustment procedure^43^, each participant received and subjectively rated five shocks on a scale from 1 (not at all painful) to 5 (very painful), allowing shock intensity to converge to a level experienced as uncomfortable, but not painful. The resulting average intensity step was 4.7 (SD: 1.7).

### Statistical analyses

Statistical analyses were performed in R (R Core Team, 2021)^47^. Wilcoxon signed-rank test and paired t-tests were performed using the *stats* package^47^. Repeated measures ANOVAs were performed using the *afex* package^48^. Spearman rank order correlations were performed using the *Hmisc* package^49^. Partial eta squared (η_p_^2^), Cohen’s d (d) or rank correlation (r) effect size estimates are reported for all relevant tests. Alpha was set at 0.05 throughout all analyses. Two-tailed tests were used unless stated otherwise. Namely, one-tailed tests were used for the directional hypotheses on the difference between threat-of-shock and safe in HR, SCR, Hits, and *d’*. Confidence intervals of the effect sizes were calculated using a bootstrapping method with the *boot* package^50^.

## Data Availability

Data and code are available on the Donders Institute repository: 10.34973/y5ev-s572.
